# Improvement of isoprene production in *Escherichia coli* by rational optimization of RBSs and key enzymes screening

**DOI:** 10.1186/s12934-018-1051-3

**Published:** 2019-01-09

**Authors:** Meijie Li, Hailin Chen, Changqing Liu, Jing Guo, Xin Xu, Haibo Zhang, Rui Nian, Mo Xian

**Affiliations:** 1 Key Laboratory of Biobased Materials, Qingdao Institute of Bioenergy and Bioprocess Technology, Chinese Academy of Sciences, No.135 Songlin Road, Qingdao, 266101 People’s Republic of China; 2 University of Chinese Academy of Sciences, Beijing, 100049 People’s Republic of China

**Keywords:** Isoprene, RBS sequence optimization, T.I.R., Metabolic engineering, Enzyme screening

## Abstract

**Background:**

As an essential platform chemical mostly used for rubber synthesis, isoprene is produced in industry through chemical methods, derived from petroleum. As an alternative, bio-production of isoprene has attracted much attention in recent years. Previous researches were mostly focused on key enzymes to improve isoprene production. In this research, besides screening of key enzymes, we also paid attention to expression intensity of non-key enzymes.

**Results:**

Firstly, screening of key enzymes, IDI, MK and IspS, from other organisms and then RBS optimization of the key enzymes were carried out. The strain utilized IDI_sa_ was firstly detected to produce more isoprene than other IDIs. IDI_sa_ expression was improved after RBS modification, leading to 1610-fold increase of isoprene production. Secondly, RBS sequence optimization was performed to reduce translation initiation rate value of non-key enzymes, ERG19 and MvaE. Decreased ERG19 and MvaE expression and increased isoprene production were detected. The final strain showed 2.6-fold increase in isoprene production relative to the original strain. Furthermore, for the first time, increased key enzyme expression and decreased non-key enzyme expression after RBS sequence optimization were obviously detected through SDS-PAGE analysis.

**Conclusions:**

This study prove that desired enzyme expression and increased isoprene production were obtained after RBS sequence optimization. RBS optimization of genes could be a powerful strategy for metabolic engineering of strain. Moreover, to increase the production of engineered strain, attention should not only be focused on the key enzymes, but also on the non-key enzymes.

**Electronic supplementary material:**

The online version of this article (10.1186/s12934-018-1051-3) contains supplementary material, which is available to authorized users.

## Background

Isoprene is an important platform chemical used for the commercial production of synthetic rubber and various other compounds, such as pesticides, medicines, oil additives, fragrances, and biofuels [1, 2]. Currently, 800,000 tons of isoprene monomer are produced annually from cracking petroleum, and over 95% of isoprene is used for rubber manufacture [3]. However, the common problems of petroleum, such as irrecoverability, fluctuating price, high energy consumption and high environmental pollution, limit sustainable supply of isoprene in future [4]. As an alternative, microbial biosynthesis of isoprene has attracted increasing attention and has been explored in the last decade [5].

**Fig. 1:**
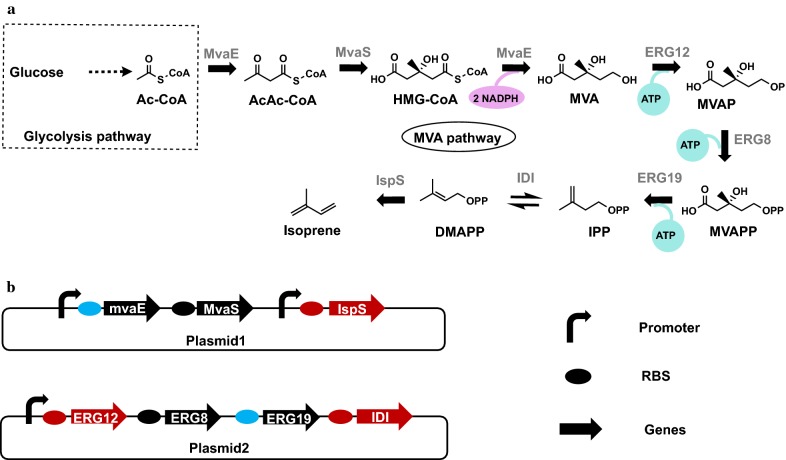
Metabolic pathway for isoprene production in engineered *E. coli*. **a** Isoprene was catalyzed by IspS from DMAPP. DAMPP was synthesized through the MVA pathway. The precursors of the MVA pathway, Ac-CoA was synthesized through the glycolysis pathway from glucose. Abbreviations: acetyl-CoA (Ac-CoA); acetoacetyl-CoA (AcAc-CoA); 3-hydroxy-3-methylglutaryl-CoA (HMG-CoA); mevalonate (MVA); mevalonate-5-phosphate (MVAP); mevalonate-5-pyrophosphate (MVAPP); isopentenyl diphosphate (IPP); dimethylallyl diphosphate (DMAPP). **b** The constructed two plasmids overexpressing genes of the MVA pathway and IspS. The promoters, RBS sequences and the genes were illustrated. The red arrow indicated the key enzyme that was modified in this research. The red oval indicated the RBS sequence that was optimized to increase the T.I.R. value. The blue oval indicated the RBS sequence that was optimized to reduce the T.I.R. value

Belonging to the isoprenoids, isoprene is synthesized by isoprene synthase (IspS) from dimethylallyl diphosphate (DMAPP), final product of mevalonate (MVA) or the methylerythritol phosphate (MEP) pathway (Fig. 1a) [6]. In order to produce isoprene in cell factory such as *Escherichia coli* or yeast, exogenous whole MVA or/and MEP pathway and IspS were overexpressed and isoprene production was detected [7, 8]. However, present isoprene productivity is far below the industry demand and improvement is required to compete with chemical production from petroleum.

The MVA pathway is commonly adopted for isoprene production (Fig. 1b). Three key enzymes, mevalonate kinase (MK/ERG12), isopentenyl-diphosphate isomerase (IDI) and IspS, impede the metabolic flux seriously. To improve isoprene production, most researches focused on these key enzymes. MK was identified as a bottleneck by targeted proteomics analysis. Higher level production of amorpha-4, 11-diene was obtained when the MK expression was up-regulated by selecting a strong promoter [9]. IDI, catalyzing the transformation between IPP and DMAPP, was proved to be another key enzyme in isoprenoids production. 1.4-fold increase of β-carotene production would be achieved by introducing a strong promoter for IDI expression [10]. In addition, summarized from the previous report, all known IspSs showed low *k*_*cat*_ and high *K*_*m*_ for DMAPP and restricted isoprene production seriously [5]. A Gal4p (a promoter) controlled expression system lead increased IspS expression and a fourfold increase of isoprene production [11]. Similar isoprene increase was achieved by enhancing expression of IspS through codon-optimization and adjustment of ribosome binding site (RBS) sequence [3]. Further isoprene production increase was obtained by inserting the MVA pathway into a high copy plasmid with a strong promoter [3]. Directed evolution has been performed for key enzymes. F310L and A570T mutations were identified after directed evolution of ISPS and a 27-fold increase of isoprene was obtained [11]. Except for the modification of the natural MVA pathway, novel pathway which circumvented the rate-limiting steps has been explored. Isoprene was synthesized from mevalonate by two steps catalyzed by OleTJE from *Jeotgalicoccus species* and OhyAEM from *Elizabethkingia meningoseptica*, shortening the MVA pathway by three steps and avoiding the three rate-limiting steps [12]. In summary, overexpression of key enzymes by selection of stronger promoter and RBS sequence and circumvention of key enzymes by a novel pathway has been usually applied for isoprene production.

On the other hand, screening the enzymes from different species with better characteristics for isoprene production was another powerful method. For isoprene production, enzymes from specific organisms were usually selected and screening enzymes from various species deserve our attention. However, little research was focused on key enzyme screening, especially the combinatorial analysis of them. Through the metabolic pathway, intracellular balance was obtained by the expression of key enzymes and non-key enzymes. However, all the engineering methods mentioned above aimed at improving expression of key enzymes and regulation of expression level of non-key enzymes was rarely reported. Moreover, when engineering methods, such as RBS sequence optimization, were applied for strains, the productivity data was usually detected; however, the change of enzyme expression was rarely analyzed.

In the present work, to increase isoprene production, we focused on the three key enzymes (MK, IDI and IspS) and non-key enzymes, acetyl-CoA acetyltransferase/HMG-CoA reductase (MvaE) and diphosphomevalonate decarboxylase (ERG19) through the whole pathway. Based on the previously constructed isoprene-producing strain [7], screening of key enzymes (MK, IDI and IspS) from different organisms and RBS sequence optimization were conducted firstly. Then enzymes with higher isoprene production were obtained and combinatorial analysis of the screened enzymes was carried out. In addition, RBS sequence optimization of non-key enzymes, MvaE and ERG19 and the effects on enzyme expression and isoprene production were examined. Furthermore, expression of key enzymes and non-key enzymes were analyzed through SDS-PAGE analysis to confirm the effect of RBS sequence optimization.

## Methods

### Strains and plasmids

*Escherichia coli* DH5α was used for gene cloning while *E. coli* Bl21(DE3) was used for expression of heterogenous genes and isoprene production. In our previous study, an engineered strain, which was defined as LMJ0 in this study, was constructed with the pYJM14, carrying the lower MVA pathway (genes ERG12, ERG8, ERG19 and IDI from *Saccharomyces cerevisiae*), and pYJM20, carrying upper MVA pathway (mvaE and mvaS genes from *Enterococcus faecalis*) and IspS_pa_ from *Populus alba* (Additional file 1: Table S1).

### Media and culture conditions

LB medium with appropriate antibiotics (100 μg/mL ampicillin or 34 μg/mL of chloramphenicol) was used for gene cloning. Modified M9 medium, adding appropriate antibiotics, was prepared as described for isoprene production under shake-flask fermentation [7].

### Constrcution of plasmids and strains

All plasmids and primers (synthesized by GENEWIZ, Suzhou) used in this study are listed in Additional file 1: Tables S1 and S2, respectively. For IDI substitution, gene IDI_bl_ (Genebank No. KND06900), IDI_bs_ (Genebank No. AIY99819), IDI_mj_ (Genebank No. WP_010870377) and IDI_sa_ (Genebank No. KII20428) were codon-optimized by JAVA Codon Adaptation Tool [13] and synthesized by GENEWIZ company. IDI_bl_ fragment was amplified by 2 × PCR Bestaq™ MasterMix (abm, Canada) using IDI_bl_-F/IDI_bl_-R, digested by *Sca* I and *Pst* I (Thermo Scientific, USA) and ligated by T4 DNA Ligase (Thermo Scientific,USA) to the linearized pYJM14 which was digested by the same enzyme, *Sac* I and *Pst* I. pT-EEE-IDI_bl_ was constructed. The other plasmids were constructed by the similar strategy, using primers listed in Table S2, correspondingly. IspS_ib_ (Genebank No. JP105673) and IspS_mp_ (Genebank No. HW399219) were analyzed by ChloroP 1.1 Server [14] to eliminate the localization sequence of chloroplastid. IspS_pa_^MT^ (L494P) and ERG12^MT^ (N66 K/I152 M) were obtained by site-directed mutagenesis (TIANGEN, Beijing), using IspS_pa_^MT^-F/IspS_pa_^MT^-R and ERG12^MT^-1-F/ERG12^MT^-1-R, ERG12^MT^-2-F/ERG12^MT^-2-R as primers (Table S2).

### Shake-flask cultures and GC analysis of isoprene

Strains were constructed by co-transformation of two plasmids into Bl21(DE3). Single colony was picked into seed culture (LB medium) and cultured at 37 °C overnight. Seed culture was transformed into 100 mL modified M9 medium in a sealed flask and cultured at 37 °C to OD_600_ of 0.6–0.8, when induction was conducted with 0.5 mM IPTG, then cultivation was continued at 30 °C for 48 h. At 3 h and 6 h after induction, 1 mL of the fermented liquid was collected by centrifugation at 5000×*g* for 15 min. The cell pellet was preserved at −20 °C for SDS-PAGE analysis. After 48 h cultivation, OD_600_ was detected. 1 mL of the gas samples from headspace of the sealed vials were analyzed by GC (Agilent 7890A, America) equipped with a flame ionization detector (FID). A HP-AL/S column (25 m × 320 μm × 8 μm) was used with nitrogen as carrier gas. The temperatures of oven, detector and injector were 50 °C, 150 °C and 50 °C, respectively.

### SDS-PAGE analysis of protein expression

The preserved cell pellet was resuspended in 100 μL lysis buffer (Beyotime, Shanghai) and then placed on ice for 1 h. Centrifugation at 5000×*g* for 10 min was conducted to separate the soluble protein and other cell fragments. Concentration of the soluble protein was detected by a BCA protein assay kit (Beyotime, Shanghai). SDS-PAGE analysis was performed to detect the protein expression.

### Data analysis

Translation initiation rate (T.I.R.) of different genes were analyzed by RBS Calculator [15, 16]. All results were expressed as mean ± standard deviation (SD). The results were analysed by OriginPro 9.0 and column charts were made. The showed figures were made by Adobe Photoshop CS5.

### Accession numbers for the various genes

The codon-optimized gene sequences were submitted into the GeneBank and the accession numbers were provided. The accession numbers of codon-optimized IDI_bl_, IDI_bs_, IDI_mj_ and IDI_sa_ were MH084474, MH084475, MH084476, MH084477. The accession numbers of codon-optimized IspS_ib_ and IspS_mp_ were MH084470 and MH084471. The accession numbers of codon-optimized MK_cv_ and MK_mm_ were MH084472 and MH084473.

## Results

In our previous study, an isoprene-producing strain was constructed through overexpressing the hybrid MVA pathway and IspS_pa_ from *P. alba* in *E. coli*. 287 mg/L isoprene production was achieved under shake flask condition [7]. MK, IDI and IspS were identified as bottlenecks through the isoprene producing pathways (Fig. 1b). Release bottlenecks through utilizing enzymes with better performance and RBS sequence optimization were conducted. In addition, RBS sequence optimization of non-key enzymes, MvaE and ERG19 were performed.

### Improvement of isoprene production through enzyme screening and enhancing RBS strength of key enzymes

**Fig. 2:**
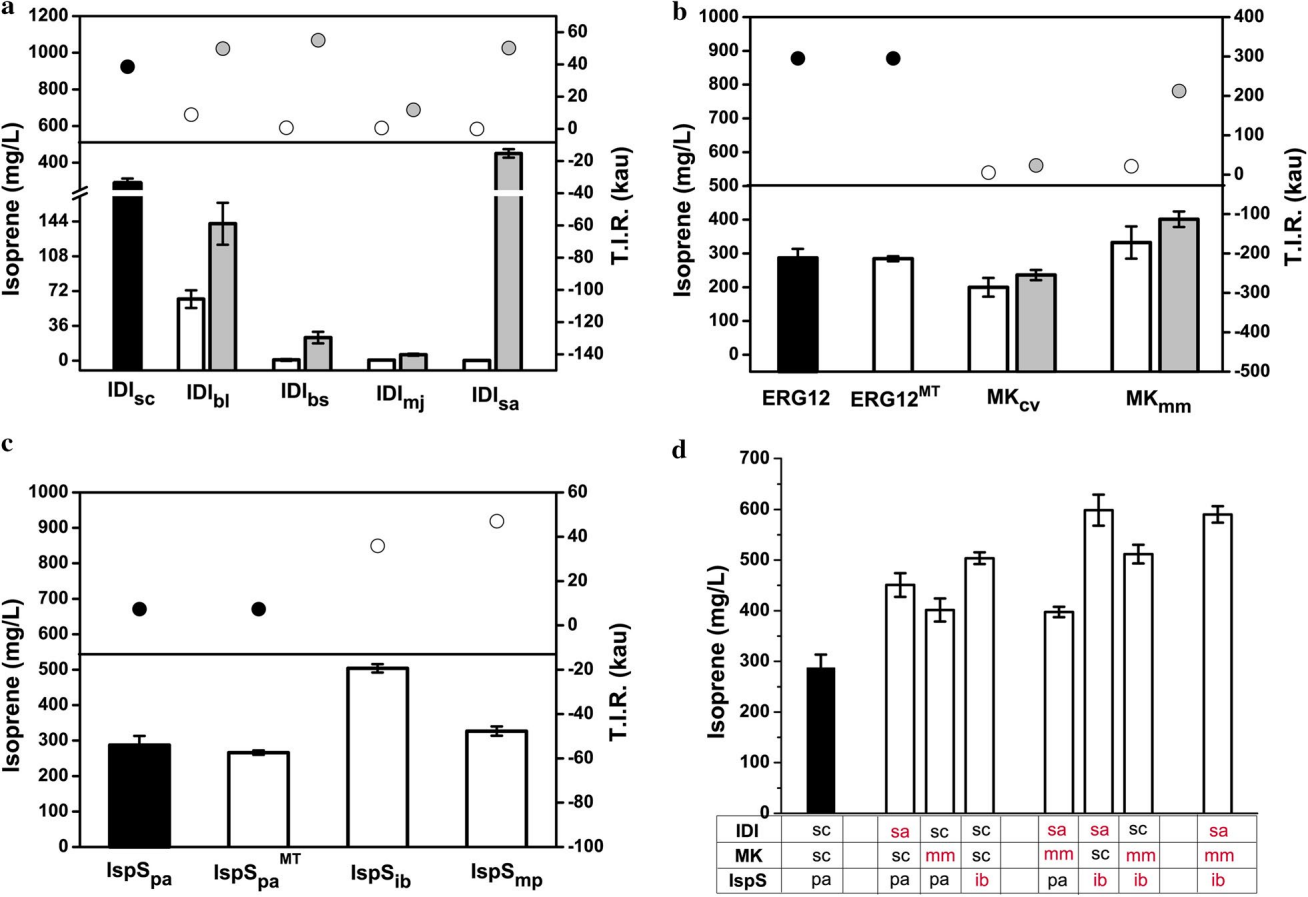
Isoprene production of strains with key enzyme modification. **a** Isoprene production of strain engineered with IDI modification and the predicated T.I.R. value of IDI. **b** Isoprene production of strain engineered with MK modification and the predicated T.I.R. value of MK. **c** Isoprene production of strain engineered with IspS modification and the predicated T.I.R. value of IspS. **d** Isoprene production of strain engineered through combination of the three enzymes, IDI_sa_, MK_mm_ and IspS_ib_. The black column indicated the isoprene production of original strain. The white column indicated that isoprene production of strain with only enzyme substitution. The gray column indicated the isoprene production of strain with enzyme substitution and RBS sequence optimization. The dot indicated the corresponding T.I.R. values. The experiment was conducted in triplicate. Bar represents mean ± s.d

IDI, catalyzing the transformation between IPP and DMAPP, is proved to be a key enzyme in partial isoprenoids production. IDI from *S. cerevisiae* (IDI_sc_) was introduced into the engineered strain in our previous study. IDI from *Bacillus subtilis* (IDI_bs_) and several Type 2 IDIs, IDI_bl_ from *Bacillus licheniformis*, IDI_mj_ from *Methanocaldococcus jannaschii* and IDI_sa_ from *Staphylococcus aureus* [17] were selected for IDI optimization. Dramatically decreased isoprene production resulted after substitution of IDI_bl_, IDI_bs_, IDI_mj_ and IDI_sa_, separately (Fig. 2a and Additional file 1: Fig. S2a, white column). Then, RBS sequence optimization was conducted. Analyzed by RBS Calculator, T.I.R. of IDI_bl_, IDI_bs_, IDI_mj_ and IDI_sa_ were 8.906 kau, 0.784 kau, 0.649 kau and 25 kau separately, far lower than IDI_sc_, 38.626 kau (Fig. 2a, white dot). It was speculated that the decreased isoprene production resulted from the weak expression of IDI, which was reflected by the low T.I.R. value. Then, RBS sequences of IDI_bl_, IDI_bs_, IDI_mj_ and IDI_sa_ were changed and relevant T.I.R.s were increased to 49.854 kau, 55.034 kau, 11.808 kau and 50.297 kau separately (Fig. 2a, gray dot). As a consequence, improved isoprene production was obtained (Fig. 2a and Additional file 1: Fig. S2a, gray column). The strain engineered with RBS sequence optimization of IDI_sa_ showed the highest production, 451 mg/L, 1.57-fold increase to the original strain and 1610-fold increase to the strain with only IDI_sa_ substitution. The OD_600_ values of different strains indicated that the strain with high isoprene production showed low growth (Additional file 1: Fig. S1a). In conclusion, great difference of isoprene production resulted from the change of RBS strength of IDI, the key enzyme of the pathway.

As a key enzyme, MK (ERG12) was demonstrated to be an essential regulatory point in MVA pathway and it is feedback inhibited by downstream intermediates, such as DMAPP, IPP, FPP and GPP. Several MKs from different organisms were proved to show preferable characteristics and were utilized in this research. A N66K/I152M mutation of ERG12 (ERG12^MT^) which showed 148% increase of specific activity and 33% decrease of feedback resistance, was reported [18]. However, the strain engineered with ERG12^MT^ in this research have the same isoprene production as the wide type (Fig. 2b and Additional file 1: Fig. S2b). MK from *Corynebacterium variabile* (MK_cv_) and MK from *Methanosarcina mazei* (MK_mm_) were also selected for MK optimization. Decreased isoprene production resulted after substitution of MK_cv_ (Fig. 2b and Fig. S2b). After MK_mm_ substitution and RBS strength enhancement, 1.4-fold increase (402 mg/L) of isoprene production was obtained (Fig. 2b, gray column) and decreased OD_600_ values was detected (Additional file 1: Fig. S1b). In conclusion, the strain with MK_mm_ and RBS strength enhancement was proved to be the better producer.

IspS is an enzyme which catalyzes the production of isoprene from the isoprenoids intermediate, DMAPP, with pyrophosphate elimination. IspS_pa_ from *P. alba* was utilized in our original strain. A positive mutant, IspS_pa_^MT^, was constructed and utilized for isoprene production. However, no improved isoprene production was detected (Fig. 2c and Additional file 1: Fig. S2c). Then IspS from *Mucuna pruriens* (IspS_mp_) and IspS from *Ipomoea batatas* (IspS_ib_) were utilized for IspS optimization. Improved isoprene production, 327 mg/L and 504 mg/L (1.9-fold increase) were achieved, separately (Fig. 2c). The OD_600_ values of different strains were showed in Additional file 1: Fig. S1c. Furthermore, RBS sequence optimization of IspS_ib_ was performed and no increased isoprene production was achieved (data not show). It is speculated that the original RBS sequence of IspS_ib_, with a 35.845 kau T.I.R. value (Fig. 2c, white dot), is strong enough for IspS_ib_ expression. Further RBS sequence optimization may result in excessive IspS expression, which may lead to reduced expression of other proteins. The unbalanced protein expression may lead to decreased isoprene production. In conclusion, 1.9-fold increase of isoprene production resulted after utilizing IspS_ib_.

Isoprene production was further improved through three screened enzymes utilization, IDI_sa_, MK_mm_ and IspS_ib_. To further improve isoprene production, combination of the three enzymes was conducted. Combinatorial optimization of IDI_sa_ and IspS_ib_ was conducted and improved isoprene production, 599 mg/L, 2.1-fold increase comparing to the original strain, was achieved, higher than the strains when IDI_sa_ and IspS_ib_ were utilized singly (Fig. 2d). However, no further increase of isoprene production resulted when additional MK_mm_ substitution was added to the strains (Fig. 2d and Additional file 1: Fig. S2d). In the strain of modified with both MK_mm_ and IspS_ib_, 512 mg/L of isoprene production was achieved, same as the strain modified with only IspS_ib_ (Fig. 2d), indicating that MK node need more modifications. The strain modified with MK_mm_ and IDI_sa_ show the same isoprene production as the strain modified with only MK_mm_ substitution and reduced production compared to the strain modified with only IDI_sa_ substitution. The OD_600_ values of different strains were showed in Additional file 1: Fig. S1d. These result indicated that when IDI_sa_ or IspS_ib_ or both of them were utilized, MK_mm_ substitution lead to no improvement of isoprene production, even decreased production, which indicated that the MK node need more research, such as RBS sequence optimization of MK_mm_ and finding new MKs from other organisms. In conclusion, the strain modified with IDI_sa_ and IspS_ib_ showed the highest isoprene production, 2.1-fold increase comparing to the original strain.

### Enhancing isoprene production through weakening RBS strength of non-key enzymes

**Fig. 3:**
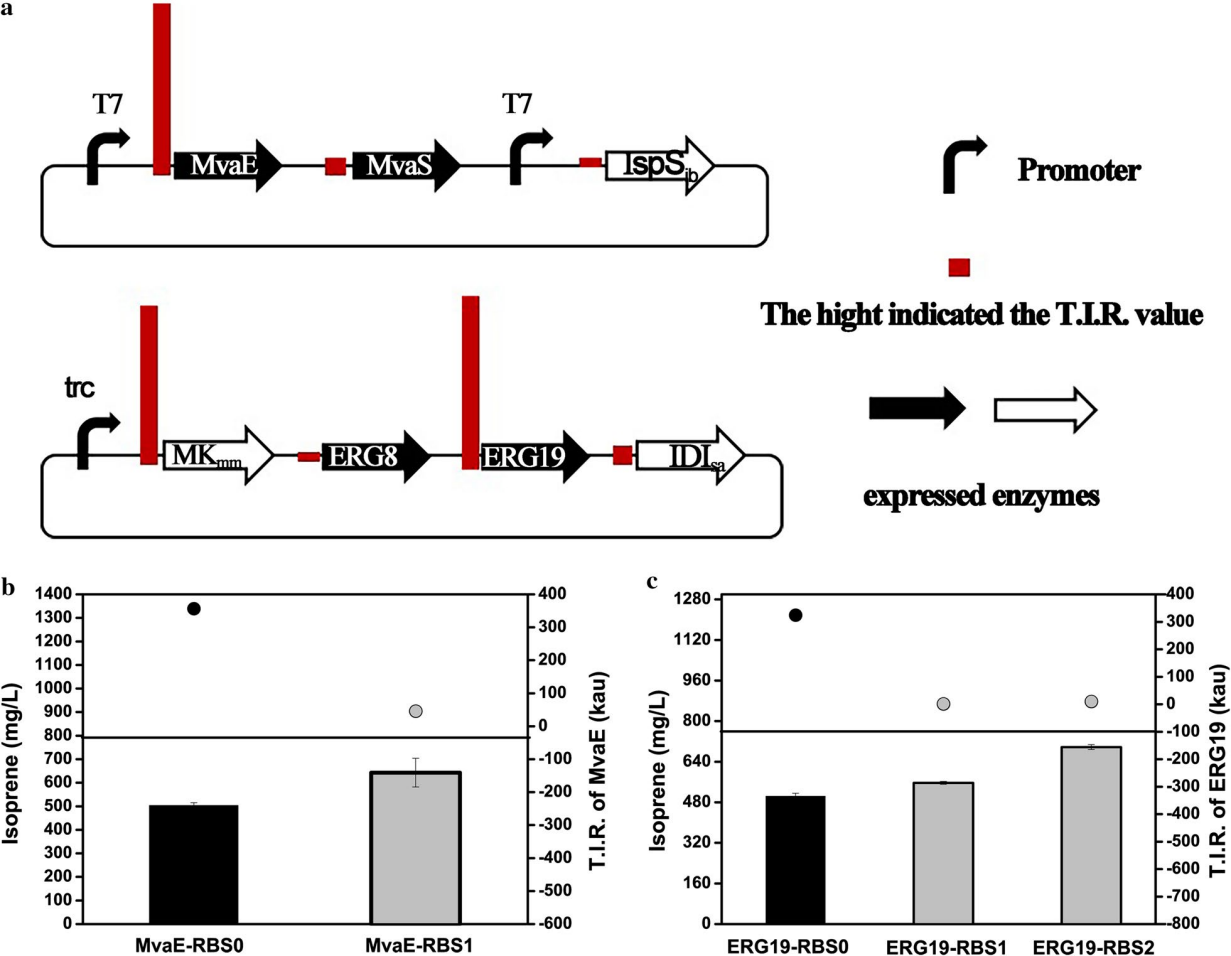
Isoprene production of strains modified with weakened RBS sequence of non-key enzymes, ERG19 and MvaE. **a** Illustration of the constructed plasmids in this study and predicted T.I.R. of every gene. The height of the red columns indicated the T.I.R. value. **b** Isoprene production of strain with modification of RBS sequence of MvaE. **c** Isoprene production of strain with modification of RBS sequence of ERG19. The black column indicated the isoprene production of the control strain. The gray column indicated the isoprene production of strain with RBS sequence optimization. The dot indicated the corresponding T.I.R. values. The experiment was conducted in triplicate. Bar represents mean ± s.d

Except for the modification of the key enzymes, RBS sequence optimization of non-key enzymes was performed and a positive result was obtained. Firstly, T.I.R. value of different genes through the whole isoprene production pathway were predicted by RBS Calculator. The T.I.R. value of MvaE and ERG19, which were not reported bo be bottlenecks, were relatively high, 357 kau and 325 kau separately, which were about 6-19 fold more than other genes, 19 kau, 39 kau, 49 kau and 36 kau for ERG8, IDI, mvaS and IspS_ib_ separately (Fig. 3a). Considering that MK (ERG12) was a key enzyme, even through the T.I.R. value of ERG12 (295 kau) was relatively high, no RBS modification was conducted. RBS sequence of MvaE and ERG19 were optimized to reduce the T.I.R. value, from 357 kau (RBS0) to 46 kau (RBS1) for MvaE, and from 325 kau (RBS0) to 1 kau (RBS1) and 9.8 kau (RBS2) for ERG19 (Fig. 3b, c). As a result, 643 mg/L of isoprene production, 1.28-fold increase to the control strain, was achieved with MvaE-RBS1 (Fig. 3b). Similarly, reducing T.I.R. of ERG19 from 324.611 kau to 9.856 kau (ERG19-RBS2), the engineered strain produced the highest amount of isoprene, 698 mg/L, 1.39-fold to the control strain and 2.6-fold to the original strain (Fig. 3c). After RBS sequence optimization of MvaE and ERG19, increased strain yields were also detected (Additional file 1: Fig. S2e, f). The OD_600_ values of strains were showed (Additional file 1: Fig. S1e, f). It is speculated that in the strain with MvaE-RBS1 and ERG19-RBS2 modification, decreased MvaE and ERG19 expression was obtained, which lead to increased expression of other key enzymes. Combining modification of T.I.R. of ERG19-RBS2 and MvaE-RBS1, increased isoprene production, 623 mg/L, comparing to the control strain was obtained, not as high as the strain with MvaE-RBS1 or ERG19-RBS2 modification singlely (data not show). The improved isoprene production indicated that reduction of T.I.R. of non-key enzymes, MvaE and ERG19, had positive effect on strain optimization.

### Desired enzyme expression after RBS sequences optimization

**Fig. 4:**
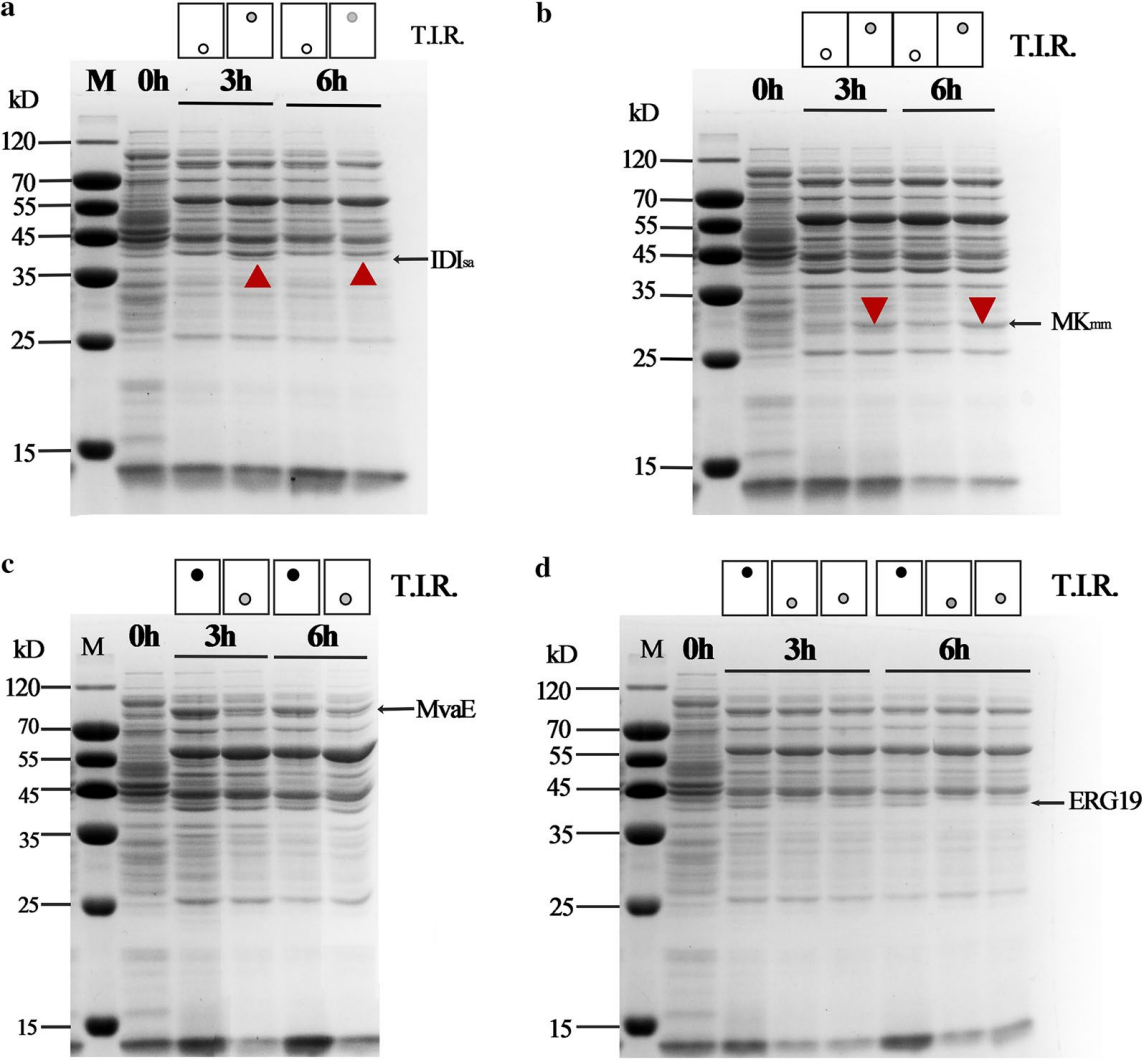
Enzyme expression of the strain with different RBS sequences and different T.I.R. value. **a** IDI_sa_ expression of the strain with different RBS sequences and different T.I.R. value. **b** MK_mm_ expression of the strain with different RBS sequences and different T.I.R. value. **c** MvaE expression of the strain with different RBS sequences and different T.I.R. value. **d** ERG19 expression of the strain with different RBS sequences and different T.I.R. value. The height of the dot indicated the T.I.R. value

RBS sequence optimization of the key enzyme and the non-key enzyme were applied to regulate enzyme expression of the pathway, which lead to the balance of the metabolic flux and incresed isoprene production. To certify the correspondence between RBS sequence strength and enzyme expression, SDS-PAGE analysis was applied to analyze enzyme expression of different strains. The strains modified with IDI_sa_ substitution, with or without RBS sequence optimization, with low or high T.I.R. value, were analyzed. Nearly no IDI_sa_ was detected before RBS sequence optimization, with low T.I.R. value (Fig. 4a). Improved IDI_sa_ expression was detected after T.I.R. value was increased through RBS sequence optimization (Fig. 4a). Similarly, the enzyme expression of strains engineered with MK_mm_ modification were conducted. The result indicated that enhanced MK_mm_ expression was obtained when T.I.R. value was increased from 21.7 to 212.8 kau (Fig. 4b). The enzyme expression of strains with the non-key enzymes, MvaE and ERG19, modification were also performed. SDS-PAGE analysis indiacted that decreased MvaE expression was detected when MvaE-RBS1 was utilized (Fig. 4c). Reduced ERG19 expression was also detected when ERG19-RBS1 and ERG19-RBS2 were utilized (Fig. 4d). The improved expression of key enzyme, MK and IDI, and the decreased expression of the non-key enzymes, MvaE and ERG19, indicated that RBS sequence optimization according to the predicated T.I.R. value was a practicable approach.

## Discussion

In this research, enzyme screening and RBS sequence optimization of key enzyme, IDI, were conducted. IDI_bs_ substitution lead to decreased isoprene production. However, in another study, introduction of IDI_bs_ into carotene-producing strain lead to improved productivity [19]. In view of the complexity of strain inner environment, the different performance of IDI_bs_ in different engineered strain was reasonable to understand. Type 2 IDI showed better activities than type 1 IDI in lycopene-producing strain [20]. IDI_sa_, a Type 2 IDI, showed better performance in this study. IDI_sa_ has been studied as a classic enzyme for catalytic mechanism analysis of type 2 IDI and the lower MVA pathway from *S. aureus* have been widely utilized for isoprenoids production [21, 22]. However, for engineering of cell factories for isoprene production, IDI_sa_ was utilized at the first time. Increased IDI_sa_ expression and isoprene production were detected simultaneously after the predicated T.I.R. value was enhanced through RBS sequence optimization. It is reasonable to speculate that more isoprene production will be obtained with further optimization of RBS sequence, considering that only two RBS sequences of IDI_sa_ were explored.

Modification of another key enzyme, MK (ERG12), was performed. A ERG12 mutation (ERG12^MT^) which show better characteristics was constructed and the unchanged isoprene production indicated that the higher enzyme activity detected in vitro did not mean better performance in vivo [18]. In another study, MK_cv_ has been attempted for isoprene production and 11.5-fold increase was achieved, even through the lower *k*_*cat*_/*K*_*m*_^*DMAPP*^ (0.05), comparing to ERG12 (*k*_*cat*_/*K*_*m*_^*DMAPP*^) [23]. Different from other MKs, activity of MK_mm_ was proved to be not inhibited by downstream intermediates [24]. Dramatically increase of isoprene production, about 11–12 fold, was obtained in strain utilized MK_mm_ and MK_cv_ [23]. However, in this study, relatively small increase was detected after modification of MK_mm_ and no increase was detected for MK_cv_. The different performance of MK in different system indicated that the production of the target product was substantially determined by the whole system, not just one or two genes, even though optimization of one gene can lead to a great difference sometimes. It is speculated that MK in this system may not be a key enzyme and optimization of MK has little effect on the metabolic flux in the engineered strain.-

IspS from gray poplar (*Populus alba* × *Populus tremula*) was isolated firstly in 2001 and IspSs from polar and kudzu were widely utilized for isoprene bio-production [25]. We summarized the previous studies for IspSs from other species and utilized the IspSs with better performance in our expression system. A L494P mutation of IspS_pa_ showed higher *k*_*cat*_ (2.1) and lower *K*_*m*_^*DMAPP*^ (3.6), comparing to the wide type, *k*_*cat*_ (1.5) and *K*_*m*_^*DMAPP*^ (7) [26]. However, in our research, isoprene production remained unchanged after utilizing L494P mutation, which indicated that high *k*_*cat*_ and low *K*_*m*_^*DMAPP*^ not always means high productivity and complicated intracellular environment cannot be changed by only one enzyme. Engineered strain with IspS_mp_ substitution was reported to have significantly increased isoprene production [27]. However, when the same IspS_mp_ was introduced into our isoprene-producing system, only subtle increase was obtained. In consideration of T.I.R. value of IspS_mp_ is the highest among all the IspSs used in this research (Fig. 2c), it is speculated that no further increase in production would obtained when T.I.R. value of IspS_mp_ is increased after RBS sequence optimization. A novel IspS from *I. batatas* (IspS_ib_) was identified through genome mining and performed better than other IspSs [28]. Similar isoprene production increase was detected in this study. IspS_ib_ show better characteristics and deserve further research.

**Fig. 5:**
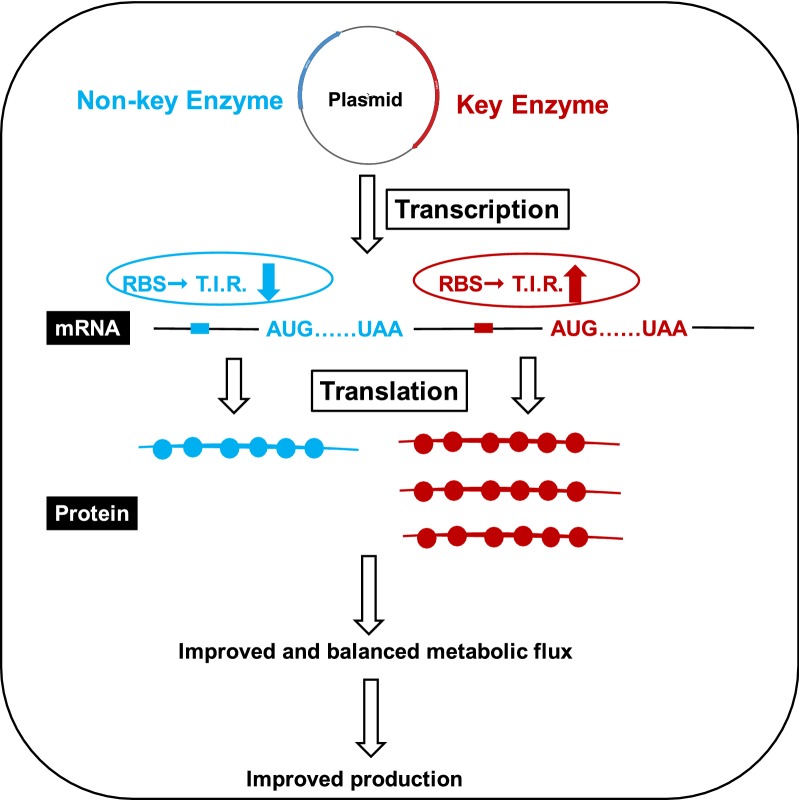
Illustration of the regulation strategies utilized in this research. Regulation of key enzyme and non-key enzyme expression through RBS sequence optimization

Eight steps and eight enzymes are required for isoprene production from acetyl-CoA and the balance of the metabolic flux is difficult to achieve. To achieve the balance of metabolic flux, regulation of enzyme expression is necessary. Many factors, including promoter, RBS sequence, temperature, PH, chaperone protein, etc., were proved to influence enzyme expression. Generally, enzyme expression was mainly regulated at transcriptional, translational, and post-translational levels. Promoter optimization, which regulates enzyme expression at the transcriptional level, was widely utilized. In the engineered isoprene-producing cell factories, three promoters with different strength, P_T7_, P_Tac_ and P_Ara_, were screened and 2.94-fold increase was achieved finally [29]. However, increased mRNA expression are not always corresponding to increased protein expression, and regulation at the transcriptional level is unstable. Furthermore, when mutiple genes are overexpressed for target product, it is unfeasible to regulate every single gene expression at the transcriptional level. At the translational level, antisense RNA was applied. To guide more DMAPP to produce IspS, antisense RNA strategies, targeting ispA, ispB and ispD, were utilized to reduce by-product production, and improved isoprene production was observed [30]. However, RNA is easily degraded in environment and strength of RNA interference is not easy to control. At the post-translational level, scaffold protein was utilized to spatially enclose the expressed enzymes in the same space [31]. The upper MVA pathway enzymes were tagged with ligands specific for the domains of the scaffold and were co-localized in the scaffold protein, leading to mevalonate production increase [32]. However, expression of scaffold protein brings extra metabolic burden to the engineered microbes. As another strategy regulating expression at the translational level, RBS sequence optimization circumvents the problem mentioned above. Considering multiple enzymes were expressed and the metabolic flux was tightly blocked in the engineered strain, RBS sequence optimization was applied to different enzymes (Fig. 5). The desired enzyme expression after RBS sequence optimization was detected in this study, not only the enhanced expression of key enzyme, but also the decreased expression of non-key enzyme. RBS sequence optimization was demonstrated to be an effective strategy to regulation enzyme expression level at the translational level. It should be noted that RBS sequence is one of the factors that affect enzyme expression, but not the only one. Furthermore, improved isoprene production was detected after RBS sequence optimization. It was reasonable to speculate that more intracellular balance, maybe more expression of bottlenecks, resulted from reduction of T.I.R. of the non-key enzymes, MvaE and ERG19, and increase of T.I.R. of key enzymes.

For strain engineering, most of the approaches can be divided into two types, rational engineering and adaptive evolution. The strategies regulating enzyme expression, including RBS sequence optimization, belong to rational engineering. For isoprene production, rational engineering approaches are mostly performed. In the last 17 years, almost thirty articles about microbial isoprene production have been published and, to our knowledge, only one research is about adaptive evolution, directed evolution of isoprene synthetase [11, 33]. RBS sequence optimization of key-enzymes and non-key enzymes was proved to be a useful strategy in this study and strain with improved isoprene production was obtained. However, RBS sequence optimization, including all the rational engineering, not always work well. Decreased isoprene production also resulted after the RBS sequence optimization of IspS_ib_ in this study. On the other hand, directed evolution of isoprene synthetase was performed and isoprene production was increased by threefold. Adaptive evolution is a powerful strategy for strain engineering, without considering the enzymes, the metabolic fluxes the products toxicity, etc. However, difficulty in high-throughput screening of isoprene limit its application [33]. When this problem was resolved, combination of rational engineering and adaptive evolution may be a better strategy for subsequent study.

In the previous study, most researches focused on the key enzymes through the whole MVA pathway and almost no articles showed any interest in non-key enzymes. However, as we mentioned above, the production of the target product was the result of the whole internal environment, which was determined not only by the key enzymes, but also by the non-key enzymes, such as MvaE and ERG19. It is the first time that down-regulation of non-key enzymes help to increase target productivity. For metabolic engineering, modification of metabolic pathway should not only focused on up-regulation of key genes, but also down-regulation of non-key genes (Fig. 5). We should analyze the RBS strength of multiple genes when engineering an strain, except for the enzyme activity and affinity.

## Conclusions

In conclusion, to enhance isoprene production of previously engineered strains, enzyme screening and RBS sequence optimization for key enzymes were firstly applied. Increased isoprene production (Table 1) was obtained and the strains engineered with IDI_sa_, IspS_ib_ or MK_mm_ performed better. IDI_sa_ was firstly tested to show better performance than other IDIs. Improved IDI_sa_ expression and 1610-fold increase of isoprene production were obtained after RBS sequence optimization. In addition, RBS strength of non-key enzymes (ERG19 and MvaE) were weakened and decreased ERG19 and MvaE expression were obtained, leading to 2.6-fold increase of isoprene production finally (Table 1). In this research, increased enzyme expression for key enzymes and decreased expression for non-key enzymes were examined after RBS sequence optimization, which indicated that regulate enzyme expression at the translational level is a powerful strategy. RBS sequence optimization of enzymes, especially the non-key enzymes, were firstly applied for isoprene production and positive results were obtained. We believe this research is helpful for building of an engineered strain, not only for isoprene production, but also for other chemicals.

**Table 1:** The steps in the metabolic engineering of *E. coli* for isoprene production in this work

Step	Optimization strategy	Strain	Plasmids	Isoprene production (mg/L)	Improvement over original strain (fold)
0	–	LMJ0	pYJM14/ pYJM20	287	–
1	Enzyme screening and RBS sequence optimization of key enzyme, IDI	LMJ8	pT-EEE-IDI_sa_-RBS/ pYJM20	451	1.57
2	Enzyme screening and RBS sequence optimization of key enzyme, MK	LMJ17	pT-EEI-MK_mm_-RBS/ pYJM20	402	1.4
3	Enzyme screening of key enzyme, IspS	LMJ11	pYJM14/ pA-MM-ispS_ib_	504	1.9
4	Combinatorial optimization of key enzymes, IDI, MK and IspS	LMJ12	pT-EE-IDI_sa_-RBS/ pA-MM-ispS_ib_	599	2.1
5	RBS sequence optimization of non-key enzyme, MvaE and ERG19	LMJ22	pT-EEI-ERG19-RBS2/ pA-MM-ispS_ib_	698	2.6

## Additional file

**Additional file 1. Table S1**. Constructed plasmids and strains in this study; **Table S2**. Primers used in this study; **Fig. S1**. OD600 of strains constructed in this study. **a**. OD600 of strain engineered with IDI modification. **b**. OD600 of strain engineered with MK modification. **c**. OD600 of strain engineered with IspS modification. **d**. OD600 ofstrain engineered through combination of the three enzymes, IDI_sa_, MK_mm_ and IspS_ib_. **e**. OD600 of strain with modification of RBS sequence of MvaE. **f**. OD600of strain with modification of RBS sequence of ERG19. The black column indicated the OD600 of original strain. The white column indicated that OD600 of strain with only enzyme substitution. The gray column indicated the OD600of strain with RBS sequence optimization. The experiment was conducted in triplicate. Bar represents mean±s.d.; **Fig. S2**. Yields of strains constructed in this study. **a**. Yields of strain engineered with IDI modification. **b**. Yields of strain engineered with MK modification. **c**. Yields of strain engineered with IspS modification. **d**. Yields of strain engineered through combination of the three enzymes, IDI_sa_, MK_mm_ and IspS_ib_. **e**. Yields of strain with modification of RBS sequence of MvaE. **f**. Yields of strain with modification of RBS sequence of ERG19. The black column indicated the isoprene yields of original strain. The white column indicated that isoprene yields of strain with only enzyme substitution. The gray column indicated the isoprene yields of strain with RBS sequence optimization. The experiment was conducted in triplicate. Bar represents mean±s.d.
