# Supplementary material for: Improvement of isoprene production in Escherichia coli by rational optimization of RBSs and key enzymes screening
Source: Microb Cell Fact. 2019 Jan 9;18:4. doi: 10.1186/s12934-018-1051-3 (PMC6327615; doi:10.1186/s12934-018-1051-3)

**Table S1. Constructed plasmids and strains in this study**

| **Names** | **Descriptions** | **References** |
| --- | --- | --- |
| Plasmids |  |  |
| pYJM14 | pTrcHis2B carrying *ERG12*, *ERG8*, *ERG19* and *IDIsc* from *S. cerevisiae* | [7] |
| pYJM20 | pACYCDuet-1 carrying *mvaE* and *mvaS* from *E. faecalis*, *IspS_pa_* from *P. alba* | [7] |
| pT-EEE-IDI_bl_ | pTrcHis2B carrying *ERG12*, *ERG8*, *ERG19* from *S. cerevisiae*, *IDI_bl_* from *B. licheniformis* | This study |
| pT-EEE-IDI_bl_-RBS | pTrcHis2B carrying *ERG12*, *ERG8* and *ERG19* from *S. cerevisiae*, *IDI_bl_* with stronger RBS sequence from *B*. *licheniformis* | This study |
| pT-EEE-IDI_bs_ | pTrcHis2B carrying *ERG12*, *ERG8* and *ERG19* from *S. cerevisiae* , *IDI_bs_* from *B. subtilis* | This study |
| pT-EEE-IDI_bs_-RBS | pTrcHis2B carrying *ERG12*, *ERG8* and *ERG19* from *S. cerevisiae* , *IDI_bs_* with stronger RBS sequence from B. subtilis | This study |
| pT-EEE-IDI_mj_ | pTrcHis2B carrying *ERG12*, *ERG8* and *ERG19* from *S. cerevisiae* , *IDI_mj_* from *M. jannaschii* | This study |
| pT-EEE-IDI_mj_-RBS | pTrcHis2B carrying *ERG12*, *ERG8* and *ERG19*9 from *S. cerevisiae* , *IDI_mj_* with stronger RBS sequence from *M. jannaschii* | This study |
| pT-EEE-IDI_sa_ | pTrcHis2B carrying *ERG12*, *ERG8* and *ERG19* from *S. cerevisiae* , *IDI_sa_* from *S. aureus* | This study |
| pT-EEE-IDI_sa_-RBS | pTrcHis2B carrying *ERG12*, *ERG8* and *ERG19* from *S. cerevisiae* , *IDI_sa_* with stronger RBS sequence from *S. aureus* | This study |
| pA-MM-IspS_pa_^MT^ | pACYCDuet-1 carrying *mvaE* and *mvaS* from *E. faecalis*, *IspS_pa_*^MT^ from *P. alba* | This study |
| pA-MM-IspS_mp_ | pACYCDuet-1 carrying *mvaE* and *mvaS* from *E. faecalis*, *IspS_mp_* from *M. pruriens* | This study |
| pA-MM-IspS_ib_ | pACYCDuet-1 carrying *mvaE* and *mvaS* from *E. faecalis*, *IspS_ib_* from *I. batatas* | This study |
| pT-EEI-ERG12^MT^ | pTrcHis2B carrying *ERG12*^MT^, *ERG8*, *ERG19* and *IDI_sc_* from *S. cerevisiae* | This study |
| pT-EEI-MK_cv_ | pTrcHis2B carrying *ERG8*, *ERG19* and *IDI_sc_* from *S. cerevisiae*, *MK_cv_* from *C. variabile* | This study |
| pT-EEI-MK_cv_-RBS | pTrcHis2B carrying *ERG8*, *ERG19* and *IDI_sc_* from *S. cerevisiae*, *MK_cv_* with stronger RBS sequence from *C. variabile* | This study |
| pT-EEI-MK_mm_ | pTrcHis2B carrying *ERG8*, *ERG19* and *IDI_sc_* from *S. cerevisiae*, *MK_mm_* from *M. mazei* | This study |
| pT-EEI-MK_mm_-RBS | pTrcHis2B carrying *ERG8*, *ERG19* and *IDI_sc_* from *S. cerevisiae*, *MK_mm_* with stronger RBS sequence from *M. mazei* | This study |
| pT-EE-IDI_sa_-MK_mm_-RBS | pTrcHis2B carrying *ERG8*, *ERG19* from *S. cerevisiae*, *MK_mm_* with stronger RBS sequence from *M. mazei*, *IDI_sa_* with stronger RBS sequence from *S. aureus* | This study |
| pT-EEI-ERG19-RBS1 | pTrcHis2B carrying *ERG12*, *ERG8*, *ERG19* with attenuated RBS strength and *IDI_sc_* from *S. cerevisiae*, | This study |
| pT-EEI-ERG19-RBS2 | pTrcHis2B carrying *ERG12*, *ERG8*, *ERG19* with attenuated RBS strength and *IDI_sc_* from *S. cerevisiae*, | This study |
| pA-M-ispS_ib_-MvaE-RBS1 | pACYCDuet-1 carrying *mvaE* with attenuated RBS strength and *mvaS* from *E. faecalis*, *IspS_ib_* from *I. batatas* | This study |
| pA-M-ispS_ib_-MvaE-RBS2 | pACYCDuet-1 carrying *mvaE* with attenuated RBS strength and *mvaS* from *E. faecalis*, *IspS_ib_* from *I. batatas* | This study |
| **Strains** |  |  |
| LMJ0 | Bl21(DE3)/pYJM14 + pYJM20 | This study |
| LMJ1 | Bl21(DE3)/ pT-EEE-IDI_bl_ + pYJM20 | This study |
| LMJ2 | Bl21(DE3)/ pT-EEE-IDI_bl_-RBS + pYJM20 | This study |
| LMJ3 | Bl21(DE3)/ pT-EEE-IDI_bs_ + pYJM20 | This study |
| LMJ4 | Bl21(DE3)/ pT-EEE-IDI_bs_-RBS + pYJM20 | This study |
| LMJ5 | Bl21(DE3)/ pT-EEE-IDI_mj_ + pYJM20 | This study |
| LMJ6 | Bl21(DE3)/ pT-EEE-IDI_mj_-RBS + pYJM20 | This study |
| LMJ7 | Bl21(DE3)/ pT-EEE-IDI_sa_ + pYJM20 | This study |
| LMJ8 | Bl21(DE3)/ pT-EEE-IDI_sa_-RBS + pYJM20 | This study |
| LMJ9 | Bl21(DE3)/ pYJM14 + pA-MM-ispS_pa_^MT^ | This study |
| LMJ10 | Bl21(DE3)/ pYJM14 + pA-MM-ispS_mp_ | This study |
| LMJ11 | Bl21(DE3)/ pYJM14 + pA-MM-ispS_ib_ | This study |
| LMJ12 | Bl21(DE3)/ pT-EEE-IDI_sa_-RBS + pA-MM-ispS_ib_ | This study |
| LMJ13 | Bl21(DE3)/ pT-EEI-ERG12^MT^ + pYJM20 | This study |
| LMJ14 | Bl21(DE3)/ pT-EEI-MK_cv_ +pYJM20 | This study |
| LMJ15 | Bl21(DE3)/ pT-EEI-MK_cv_-RBS + pYJM20 | This study |
| LMJ16 | Bl21(DE3)/ pT-EEI-MK_mm_ + pYJM20 | This study |
| LMJ17 | Bl21(DE3)/ pT-EEI-MK_mm_-RBS+ pYJM20 | This study |
| LMJ18 | Bl21(DE3)/pT-EE-IDI_sa_-MK_mm_-RBS + pA-MM-ispS_ib_ | This study |
| LMJ19 | Bl21(DE3)/pYJM14 + pA-M-ispS_ib_-MvaE-RBS1 | This study |
| LMJ20 | Bl21(DE3)/pYJM14 + pA-M-ispS_ib_-MvaE-RBS2 | This study |
| LMJ21 | Bl21(DE3)/pT-EEI-ERG19-RBS1 + pA-MM-ispS_ib_ | This study |
| LMJ22 | Bl21(DE3)/pT-EEI-ERG19-RBS2 + pA-MM-ispS_ib_ | This study |
| LMJ23 | Bl21(DE3)/pT-EEI-ERG19-RBS2 + pA-M-ispS_ib_-MvaE-RBS2 | This study |

**Table S2. Primers used in this study**

| **Primers** | **5’-3’** |
| --- | --- |
| IDI_bl_-F | TAGGAGCTCAAAAAAAATGATGGTTACCCGCGCCAA |
| IDI_bl_-R | CAGCTGCAGTTAGCGCTTGCTAT |
| IDI_bl_-RBS-F | CGCCCTTAGGAGCTCACCCACTAGAAACGATAGAAAGAGAGAGGCAGAATGATGGTTACCCGCGCC |
| IDI_bl_-RBS-R | TGGTACCAGCTGCAGTTAGCGCTTGCTATACAC |
| IDI_bs_-F | TAGGAGCTCAAAAAAAATGACCCGTGCCGAGCGTAAACG |
| IDI_bs_-R | CAGCTGCAGTTAGCGCACGCTAT |
| IDI_bs_-RBS-F | CGCCCTTAGGAGCTCCGCACCGAACAACGAGAAAAATAAGGAGGTACCTATGACCCGTGCCGAGCGTAAACG |
| IDI_bs_-RBS-R | TGGTACCAGCTGCAGTTAGCGCACGCTATAACTG |
| IDI_mj_-F | TAGGAGCTCAAAAAAAATGGTGAATAATCGCAACGA |
| IDI_mj_-R | CAGCTGCAGTTATTTCAGACGCT |
| IDI_mj_-RBS-F | CGCCCTTAGGAGCTCAGATATTACAAAACCCAATAACGAGGAAAATATGGTGAATAATCGCAACGAG |
| IDI_mj_-RBS-R | TGGTACCAGCTGCAGTTATTTCAGACGCTGGCTGATC |
| IDI_sa_-F | TAGGAGCTCAAAAAAAATGAGCGATTTCCAGCGCGA |
| IDI_sa_-R | CAGCTGCAGTTAACCGCGATGGA |
| IDI_sa_-RBS-F | CGCCCTTAGGAGCTCAACAAGTATCAAAATAGGTAGGAGGACATTATGAGCGATTTCCAGCGCGAACAGC |
| IDI_sa_-RBS-R | TGGTACCAGCTGCAGTTAACCGCGATGGATATTCAG |
| IspS_ib_-F | GATATACATATGAGTAGCGCCCAG |
| IspS_mp_-F | GATATACATATGAGTGCCGTTAGC |
| IspS-R | GCCGGCAGATCTTTA |
| IspS_pa_^MT^-F | AACTGGGTGGTAGCCCGTTCGCGAAA |
| IspS_pa_^MT^-R | GGGCTACCACCCAGTTTTTCCTTGTT |
| MK_cv_-F | TGTATCGATTAAATAAGGAGGAATAAACCATGGCCCCGCATGTGGGTCA |
| MK_cv_-R | TGCAGGCCTATCGCAAATTAGCTTATTGCATCACTTCACCGG |
| MK_cv_-RBS-F | TATTAATGTATCGATCAATTCAAGATATAAGGAGATTAGAGAATGGCCCCGCATGTGGGTCATG |
| MK_cv_-RBS-R | TAAGGGTGCAGGCCTTTATTGCATCACTTCACCGG |
| MK_mm_-F | TGTATCGATTAAATAAGGAGGAATAAACCATGGTTTCTTGTTCTGCTCC |
| MK_mm_-R | TGCAGGCCTATCGCAAATTAGCTTAATCCACTTTCAGGCCC |
| MK_mm_-RBS-F | TATTAATGTATCGATACGCGAACGCGTACAATTAAGTTCGGAGGTTTCGTATGGTTTCTTGTTCTGCTCCTG |
| MK_mm_-RBS-R | TAAGGGTGCAGGCCTTTAATCCACTTTCAGGCCC |
| ERG12^MT^-1-F | GGCTCAAGCGCCTCTATGTCTGTATCAC |
| ERG12^MT^-1-R | CATAGAGGCGCTTGAGCCCAACCCAGCA |
| ERG12^MT^-2-F | GGCTCAAGCGCCTCTATGTCTGTATCACTGGCCTTAGCTA |
| ERG12^MT^-2-R | TAGCTAAGGCCAGTGATACAGACATAGAGGCGCTTGAGCC |
| ERG19-RBS1-F | GATAAATAACTCGAGATCGATTATACCGGACAACAGCAAGGATATTAGG ATGACCGTTTACACAGCATC |
| ERG19-RBS2-F | GATAAATAACTCGAGTAACGTACGGTACCAGATTAATAGGAGGCTCGAATGACCGTTTACACAGCATC |
| ERG19-RBS-R | ATTTTTTTTGAGCTCCTAAGGGCG |
| MvaE-RBS1-F | GGAGATATACCATGGCGCCAACGAACATTTACTACAATAGAGGAGACCTAATGAAAACAGTAGTTATTATTG |
| MvaE -RBS2-F | GGAGATATACCATGGAACAACACGCATACAATAAAAGGAGGCAACACAAGGATGAAAACAGTAGTTATTATTG |
| MvaE -RBS-R | CTCGAATTCGGATCCTTATTG |
|  |  |

**Fig. S1** OD600 of strains constructed in this study. **a.** OD600 of strain engineered with IDI modification. **b.** OD600 of strain engineered with MK modification. **c.** OD600 of strain engineered with IspS modification. **d.** OD600 of strain engineered through combination of the three enzymes, IDI_sa_, MK_mm_ and IspS_ib_. **e.** OD600 of strain with modification of RBS sequence of MvaE. **f**. OD600 of strain with modification of RBS sequence of ERG19. The black column indicated the OD600 of original strain. The white column indicated that OD600 of strain with only enzyme substitution. The gray column indicated the OD600 of strain with RBS sequence optimization. The experiment was conducted in triplicate. Bar represents mean±s.d.


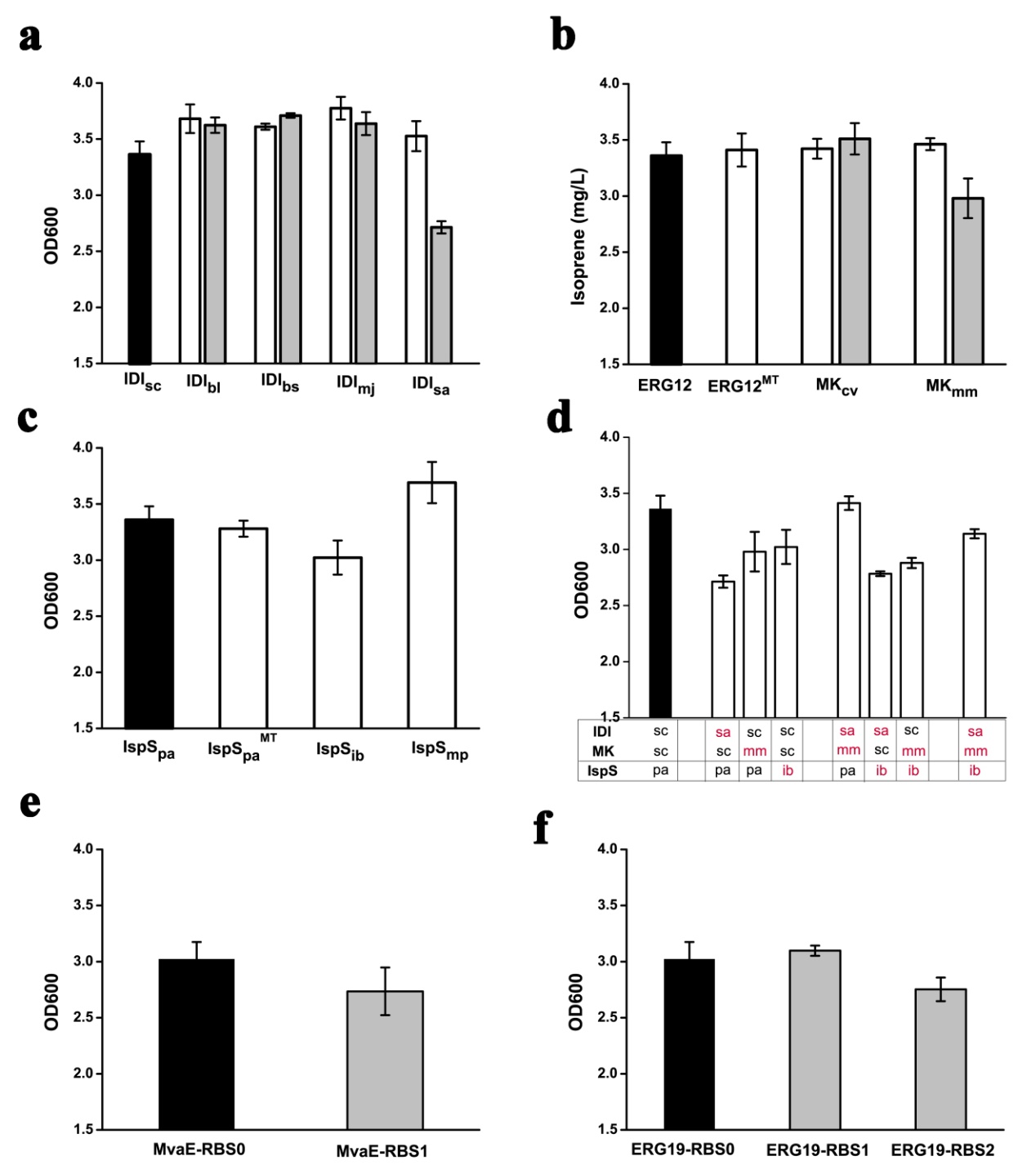


**Fig. S2** Yields of strains constructed in this study. **a.** Yields of strain engineered with IDI modification. **b.** Yields of strain engineered with MK modification. **c.** Yields of strain engineered with IspS modification. **d.** Yields of strain engineered through combination of the three enzymes, IDI_sa_, MK_mm_ and IspS_ib_. **e.** Yields of strain with modification of RBS sequence of MvaE. **f**. Yields of strain with modification of RBS sequence of ERG19. The black column indicated the isoprene yields of original strain. The white column indicated that isoprene yields of strain with only enzyme substitution. The gray column indicated the isoprene yields of strain with RBS sequence optimization. The experiment was conducted in triplicate. Bar represents mean±s.d.


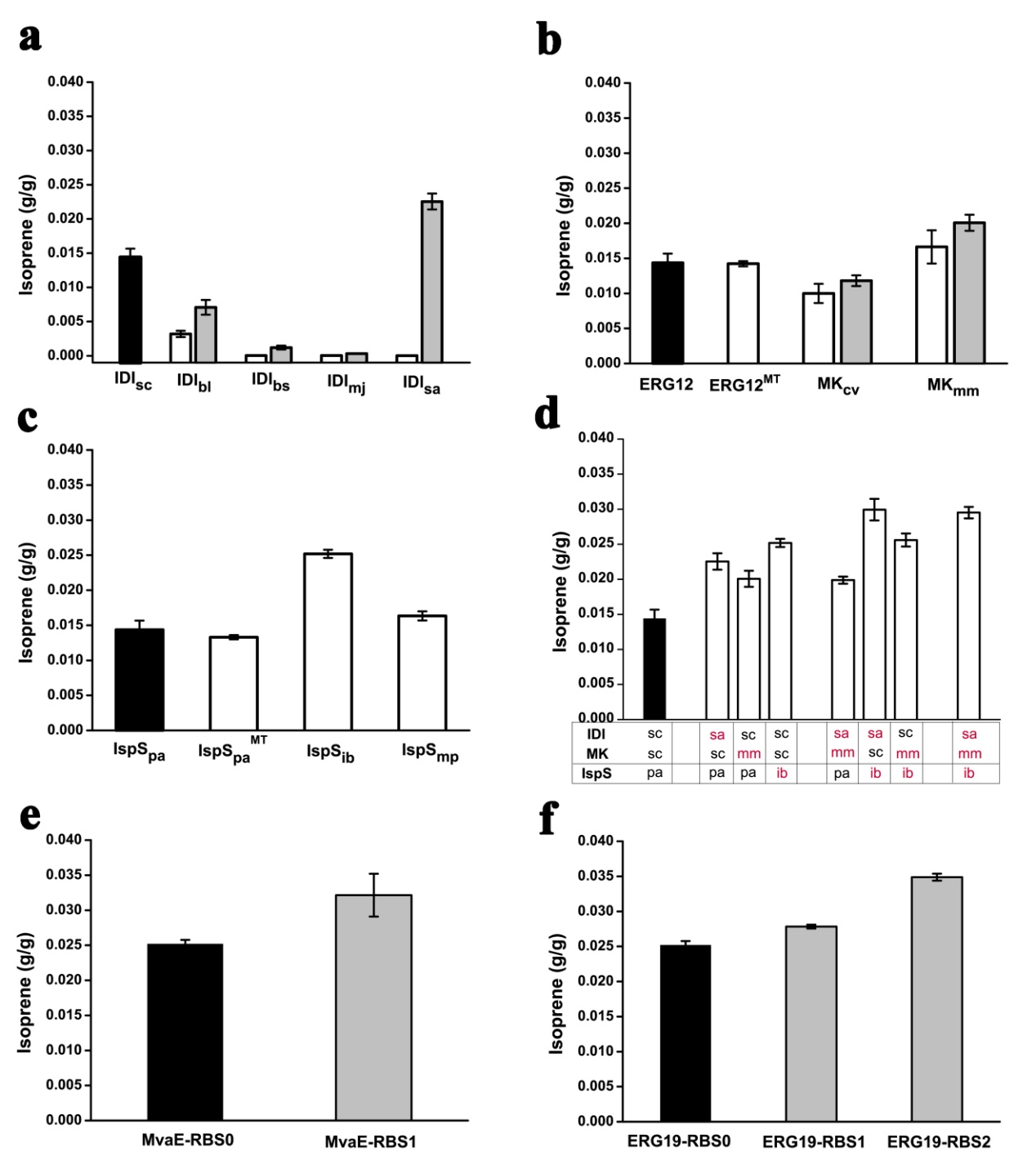

Supplement: Supplementary file 1 — Additional file 1. Table S1. Constructed plasmids and strains in this study; Table S2. Primers used in this study; Fig. S1. OD600 of strains constructed in this study. a. OD600 of strain engineered with IDI modification. b. OD600 of strain engineered with MK modification. c. OD600 of strain engineered with IspS modification. d. OD600 ofstrain engineered through combination of the three enzymes, IDIsa, MKmm and IspSib. e. OD600 of strain with modification of RBS sequence of MvaE. f. OD600of strain with modification of RBS sequence of ERG19. The black column indicated the OD600 of original strain. The white column indicated that OD600 of strain with only enzyme substitution. The gray column indicated the OD600of strain with RBS sequence optimization. The experiment was conducted in triplicate. Bar represents mean±s.d.; Fig. S2. Yields of strains constructed in this study. a. Yields of strain engineered with IDI modification. b. Yields of strain engineered with MK modification. c. Yields of strain engineered with IspS modification. d. Yields of strain engineered through combination of the three enzymes, IDIsa, MKmm and IspSib. e. Yields of strain with modification of RBS sequence of MvaE. f. Yields of strain with modification of RBS sequence of ERG19. The black column indicated the isoprene yields of original strain. The white column indicated that isoprene yields of strain with only enzyme substitution. The gray column indicated the isoprene yields of strain with RBS sequence optimization. The experiment was conducted in triplicate. Bar represents mean±s.d. [file 12934_2018_1051_MOESM1_ESM.docx]
